# Exercise interventions for depressive symptoms in adults with lung and digestive cancer: a meta-analysis of randomized controlled trials

**DOI:** 10.3389/fpsyt.2026.1833619

**Published:** 2026-06-01

**Authors:** Xiao-xia Shang, Miao Liu, Wei-ming Yang, Zheng Zhang

**Affiliations:** 1 Department of Physical Education, Hebei University of Economics and Business, Shijiazhuang, China; 2 Department of Physical Education, Tianjin Foreign Studies University, Tianjin, China; 3 Yanching Institute of Technology, Langfang, China; 4 Department of Sports Science, Kyonggi University, Suwon, Republic of Korea

**Keywords:** depressive symptoms, digestive cancer, exercise, lung cancer, meta-analysis, randomised controlled trials

## Abstract

**Background:**

Depressive symptoms are common among patients with cancer and can substantially impair quality of life, treatment adherence and overall well-being. Although exercise has been increasingly recognised as a promising non-pharmacological strategy for alleviating depression in oncology settings, existing evidence has focused predominantly on breast cancer, with limited attention to lung and digestive cancers. This meta-analysis aimed to evaluate the effects of exercise interventions on depressive symptoms in adults with lung and digestive cancer.

**Methods:**

A systematic search was conducted in PubMed, Web of Science, Embase, Cochrane Library and Scopus from database inception to March 2026. Randomized controlled trials investigating the effects of exercise interventions on depressive symptoms in adults with lung or digestive cancer were included. The primary outcome was depressive symptoms measured using validated instruments. Subgroup analyses were performed according to intervention format, exercise type and training frequency. Risk of bias was assessed using the Cochrane Risk of Bias tool version 1.

**Results:**

Eight randomized controlled trials were included in the meta-analysis. Baseline analysis showed no significant difference in depressive symptoms between the exercise and control groups before intervention. Post-intervention meta-analysis demonstrated that exercise significantly reduced depressive symptoms compared with control conditions (SMD = -0.45, P = 0.02), Although substantial heterogeneity was observed. Individually delivered programmes, walking-based exercise and moderate-frequency training (3–5 times per week) showed numerically larger effect estimates.

**Conclusions:**

Exercise interventions may reduce depressive symptoms in adults with lung and digestive cancer and represent a promising adjunctive strategy for psychological care in these populations. Although subgroup differences were not statistically significant, certain intervention characteristics may be associated with greater benefit. Further large-scale, high-quality randomized trials are needed to confirm these findings and to establish the optimal exercise prescription for reducing depressive symptoms in adults with lung and digestive cancer.

**Systematic review registration:**

https://www.crd.york.ac.uk/prospero/, identifier CRD420261336578.

## 1 Background

Depression is one of the most prevalent mental disorders worldwide and remains a major contributor to disability and reduced quality of life (1). The World Health Organization estimates that depressive disorder affects approximately 5.7% of adults globally, underscoring its substantial public-health burden (2). This burden may be even greater in oncology settings, where the psychological impact of diagnosis, treatment toxicity, symptom distress and uncertainty about prognosis frequently converge (3, 4). Among patients with cancer, depressive symptoms have been associated with impaired functioning, poorer treatment adherence and diminished quality of life (5). Adults with lung and digestive cancers may be particularly vulnerable. Lung cancer is commonly accompanied by high symptom burden, functional decline and poor prognosis, all of which may intensify emotional distress; prior studies have reported a substantial prevalence of depressive symptoms in this population (6, 7). Similarly, digestive cancers, including gastrointestinal and hepatobiliary malignancies, often involve persistent nutritional, gastrointestinal and treatment-related complications that can profoundly disrupt daily life, and systematic evidence suggests that depressive symptoms are also common in these patients (8, 9). Together, these observations support a focused examination of depressive symptoms in adults with lung and digestive cancers, rather than treating all cancer populations as a clinically homogeneous group.

Exercise has emerged as a promising non-pharmacological strategy for alleviating depressive symptoms (10). In the general population, structured physical activity has been associated with meaningful reductions in depression severity, and in oncology care, exercise is increasingly recognised as an important component of supportive management (11, 12). The 2023 American Society of Clinical Oncology guideline for adult cancer survivors recommends structured physical activity and exercise among the treatment options for depressive symptoms, reflecting the growing clinical relevance of this approach (13). Mechanistically, exercise may improve mood through intertwined biological and behavioural pathways, including reductions in inflammation, improvements in physical functioning, restoration of self-efficacy and increased social engagement (14, 15). In patients with cancer, accumulating evidence indicates that exercise can improve a range of psychosocial outcomes, including depression and anxiety; however, the strongest and most consistent evidence has largely come from breast cancer populations (16). Recent reviews in breast cancer survivors have shown that exercise can significantly reduce depressive symptoms, reinforcing the plausibility of exercise as an adjunctive strategy for cancer-related psychological distress (17).

Despite these advances, important evidence gaps remain. Much of the existing literature has pooled heterogeneous cancer types together or has focused predominantly on breast cancer survivors, limiting the interpretability and clinical applicability of the findings for other malignancies (18, 19). Lung and digestive cancers differ substantially from breast cancer in symptom profile, treatment burden, disease trajectory and rehabilitation needs, and these distinctions may influence both the baseline severity of depressive symptoms and the response to exercise interventions. Although some recent studies have begun to explore exercise and psychological outcomes in lung cancer (20), evidence remains limited, and data for digestive cancers are even more sparse. Consequently, it is still unclear whether exercise confers comparable antidepressant benefits in adults with lung and digestive cancers, and whether the currently available randomized evidence is sufficient to support subtype-specific conclusions. A meta-analysis focused on randomized controlled trials in these two cancer populations is therefore warranted to synthesise the available evidence, quantify the effect of exercise on depressive symptoms and clarify the current state of the field.

## 2 Meta-analysis results

This meta-analysis was performed according to the Preferred Reporting Items for Systematic Reviews and Meta-Analysis statement and the Cochrane Collaboration Handbook. The protocol was registered on PROSPERO (CRD420261336578).

### 2.1 Data sources and searches

A systematic literature search was conducted to identify randomized controlled trials evaluating the effects of exercise interventions on depressive symptoms in adults with lung and digestive cancer. The following electronic databases were searched from their inception to March 2026: PubMed, Web of Science, Embase and Cochrane Library. To ensure a comprehensive retrieval of relevant studies, the search strategy combined terms related to depression, exercise and cancer, using both controlled vocabulary and free-text keywords where appropriate. The core search concepts included depressive symptoms or depression, physical activity or exercise, and cancer or neoplasm. The detailed search strategies for each database are provided in **Supplementary Material 1**.

In addition to database searching, the reference lists of eligible studies and relevant reviews were manually screened to identify any additional records that might have been missed in the electronic search. No restrictions were imposed at the initial search stage regarding publication year. The final search was completed in March 2026. Where full texts were not readily accessible or where key outcome data were incomplete, attempts were made to contact the corresponding authors for clarification where feasible.

### 2.2 Inclusion and exclusion

Studies were considered eligible according to the PICOS framework. Population (P): We included studies involving adults diagnosed with lung cancer or digestive cancer. Digestive cancer was broadly defined to include malignancies of the gastrointestinal and hepatobiliary systems, such as oesophageal, gastric, colorectal, liver, pancreatic and related cancers. Studies enrolling mixed cancer populations were eligible only if data for participants with lung or digestive cancer could be extracted separately; Intervention (I): Eligible interventions were structured exercise programmes, including but not limited to aerobic exercise, resistance training, combined exercise, walking-based programmes, mind–body exercise and other planned physical activity interventions delivered in any setting. Interventions were required to include exercise as the principal component; Comparator (C): Control conditions included usual care, wait-list control, attention control, health education or other non-exercise interventions; Outcomes (O): Studies were required to report depressive symptoms or depression-related outcomes assessed using validated measurement instruments. When multiple depression-related outcomes were reported, data were extracted preferentially from the most widely used or clinically relevant scale; Study design (S): Only randomized controlled trials were included. Parallel-group and other randomized designs were considered eligible if sufficient outcome data were available for quantitative synthesis.

Studies were excluded if they: (1) involved paediatric or adolescent populations; (2) were non-randomized studies, observational studies, quasi-experimental studies, case reports, conference abstracts, review articles, study protocols or animal studies; (3) did not include an exercise-based intervention as the main treatment component; (4) did not report extractable data on depressive symptoms; (5) enrolled general cancer populations without separate data for lung or digestive cancer; or (6) were duplicate publications, in which case the most complete or most recent report was retained.

For trials with multiple eligible exercise arms sharing a common control group, relevant intervention arms were combined where conceptually appropriate to avoid double-counting of the control group. When combination was not appropriate, the shared control group was divided proportionally in accordance with Cochrane recommendations.

### 2.3 Assessment of risks of bias

The methodological quality of the included studies was assessed using the Cochrane Risk of Bias tool version 1 (RoB 1). Although RoB 2 is the current Cochrane tool for assessing risk of bias in randomized trials, RoB 1 was retained in the present review because it remains widely used in published meta-analyses of exercise-based interventions and provides a transparent domain-based assessment of key methodological issues. In addition, several included trials provided limited methodological reporting details; therefore, RoB 1 allowed a consistent and comparable assessment across all included studies.

RoB 1 evaluates potential sources of bias across the following domains: random sequence generation, allocation concealment, blinding of participants and personnel, blinding of outcome assessment, incomplete outcome data, selective outcome reporting, and other sources of bias. Each domain was judged as low risk, high risk, or unclear risk of bias, in accordance with the guidance provided in the Cochrane Handbook. The risk-of-bias assessment was conducted independently by two reviewers (SSX and LM). Any discrepancies were discussed and resolved through consultation with a third reviewer (YWM), who made the final judgement when consensus could not be reached.

### 2.4 Data extraction

Data extraction was performed independently by two reviewers (SSX and LM) using a predefined data extraction form. The following information was collected from each included study: first author, publication year, sample characteristics, cancer type, sample size, participant age, details of the exercise intervention and comparator, duration and frequency of the intervention, depression-related outcome measures, and outcome data required for the meta-analysis.

When studies reported depression outcomes using more than one assessment tool or at multiple time points, the most relevant data were selected according to the predefined review criteria. If essential data were incomplete or unclear, the corresponding study report was examined in detail to obtain the most comprehensive information possible. Any discrepancies in data extraction between the two reviewers were resolved through discussion, and unresolved disagreements were adjudicated by a third reviewer (YWM), who made the final decision.

### 2.5 Assessment of overall effect size

Meta-analyses were conducted using Review Manager (RevMan, version 5.4). Because depressive symptoms were assessed using different validated instruments across studies, including the Hospital Anxiety and Depression Scale (HADS), Patient Health Questionnaire-9 (PHQ-9), and Self-Rating Depression Scale (SDS), the standardized mean difference (SMD) with 95% confidence intervals (CIs) was used to estimate the pooled effect size. This approach allowed results measured on different scales to be combined within a common metric. Effect sizes were calculated using post-intervention mean scores and standard deviations of depressive symptoms in the exercise and control groups. No change-from-baseline values were used in the primary meta-analysis.

For all analyses, effect sizes were calculated such that a lower score indicated fewer depressive symptoms in the exercise group relative to the control group. Statistical heterogeneity among studies was assessed using the Cochran Q test and the I² statistic. An I² value greater than 50% was considered to indicate substantial heterogeneity. A random-effects model was applied when substantial heterogeneity was present; otherwise, a fixed-effect model was used. Statistical significance was defined as a two-sided P value < 0.05.

### 2.6 Subgroup analysis of exercise intervention programmes

To explore potential sources of between-study variability, prespecified subgroup analyses were conducted according to exercise type, training frequency, and intervention format. Given the small number of included trials, all subgroup analyses were considered exploratory and hypothesis-generating rather than confirmatory.

For exercise type, interventions were classified according to their principal form, including walking-based exercise, mind–body exercise, and structured exercise. For training frequency, studies were grouped according to the weekly frequency of exercise delivery, namely low frequency (≤2 times/week), moderate frequency (3–5 times/week), and high frequency (≥6 times/week). For intervention format, exercise programmes were categorised as individual-based or group-based, depending on whether the intervention was delivered primarily on a one-to-one basis or in a collective setting.

Subgroup analyses were used to examine whether effect estimates varied across intervention characteristics. However, no definitive conclusions regarding the superiority of any exercise type, training frequency, or intervention format were drawn unless supported by statistically significant between-subgroup differences.

### 2.7 Certainty of evidence assessment

The certainty of evidence for the primary outcome was assessed using the GRADE approach. The assessment considered five domains: risk of bias, inconsistency, indirectness, imprecision, and publication bias. The certainty of evidence was rated as high, moderate, low, or very low. Because depressive symptoms were the primary outcome of this review, GRADE assessment was conducted for this outcome.

## 3 Result

### 3.1 Search process

The literature search identified 1,434 records from electronic databases, including 345 from Embase, 202 from PubMed, 67 from Web of Science and 850 from the Cochrane Library. After removal of 278 duplicate records, 1,156 records remained for title screening. Of these, 1,009 records were excluded on the basis of title, and a further 33 records were excluded because they were meta-analyses or systematic reviews.

**Figure 1:**
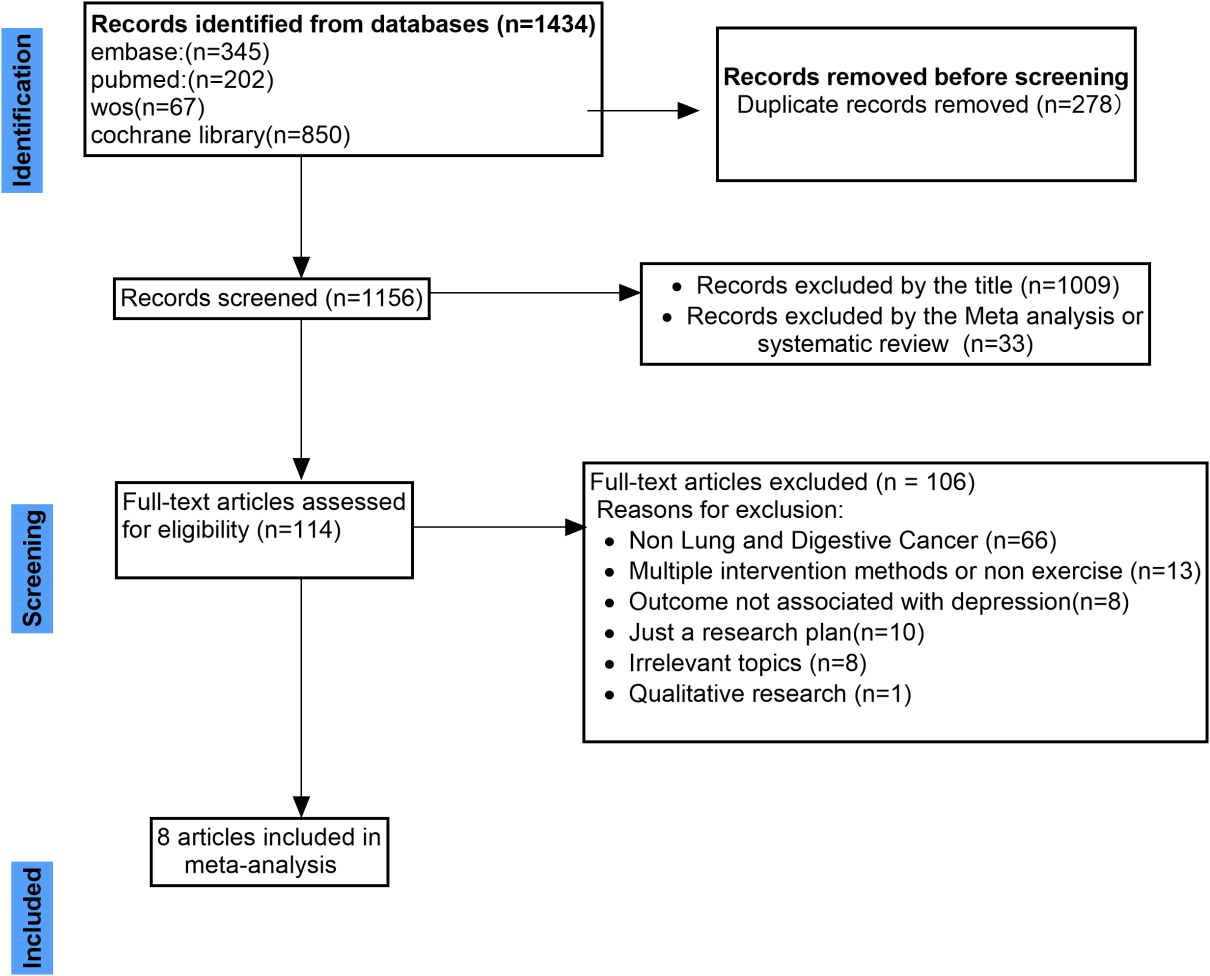
Flowchart and selection of studies.

The full texts of the remaining 114 articles were assessed for eligibility. Following full-text review, 106 articles were excluded for the following reasons: the study population did not involve lung or digestive cancer (n = 66); the intervention included multiple components or was not exercise-based (n = 13); the outcome was not related to depression (n = 8); the article was a study protocol or research plan (n = 10); the topic was irrelevant to the review question (n = 8); or the study used a qualitative design (n = 1). Ultimately, 8 articles met the inclusion criteria and were included in the meta-analysis. The study selection process is presented in **Figure 1**.

### 3.2 Characteristics of the included studies and participants

The characteristics of the 8 studies included in this meta-analysis are summarised in **Table 1**. Detailed information is provided on sample size, mean participant age, intervention and control group design, type of exercise intervention, intervention duration and frequency, as well as the depression outcome measures used in each study.

**Table 1:** Characteristics of the included studies and participants.

Studies	Sample size (IG/CG)	Age range (IG/CG)	IG type	Frequency/duration	Outcome measures
Bade, B. C. 2021	20:20	66.55 ± 7.28/63.20 ± 9.80	walking	2 times weekly/ 12 weeks	PHQ-9
Chen, H. M. 2015	50:51	64.76 ± 11.28/ 63.57 ± 10.54	walking	3 times weekly/ 12 weeks	HADS
Cheung, D. S. T. (1) 2021	10:11	61.00 ± 12.1/58.36 ± 9.3	walking	2 times weekly/ 12 weeks	HADS
Cheung, D. S. T. (2) 2021	9:11	61.11 ± 7.01/ 58.36 ± 9.32	Taiji	2 times weekly/ 12 weeks	HADS
Quist, M. 2020	110:108	65.2 ± 8.2/63.5 ± 8.7	structured exercise	2 times weekly/ 12 weeks	HADS
Rui-Chen, Ma 2021	34:35	56.97 ± 7.09/ 54.91 ± 10.09	structured exercise	7 times weekly/ 2 weeks	HADS
Ho, M. 2020	56:56	66.6 ± 9.5/ 64.9 ± 9.4	structured exercise	5 times weekly/ 12 months	HADS
Kim, J. Y. 2019	30:28	56.8 ± 10.2/ 55.7 ± 8.7	structured exercise	7 times weekly/ 12 weeks	PHQ-9
Yang, L. H. 2021	40:40	Not reported	Qigong	5 times weekly/ 4 weeks	SDS

IG, Intervention Group; CG, Control Group; HADS, Hospital anxiety and depression scale; PHQ-9, Patient Health Questionnaire-9; SDS, Self-rating depression scale.

### 3.3 Risks of bias

Risk of bias was assessed using the Cochrane Risk of Bias tool version 1, and the detailed study-level judgements are presented in **Supplementary Material 2**. Overall, the included trials showed a mixed methodological profile, with stronger performance in sequence generation and incomplete outcome data, but greater uncertainty in allocation concealment, outcome assessment and other potential sources of bias.

Across the eight studies included in the risk-of-bias assessment, all eight were judged to be at low risk of bias for random sequence generation. For allocation concealment, six studies were rated as low risk and two as unclear risk. By contrast, all eight studies were judged to be at high risk of bias for blinding of participants and personnel, which is not unexpected in exercise-based interventions where participant blinding is often impractical.

For blinding of outcome assessment, the judgements were more variable: three studies were rated as low risk, three as high risk and two as unclear risk. With respect to incomplete outcome data, all eight studies were considered to be at low risk of bias. For selective reporting, four studies were judged as low risk and four as unclear risk. Finally, for other bias, one study was rated as low risk, whereas the remaining seven studies were judged as unclear risk. Taken together, these findings suggest that the main methodological concerns in the included literature were related to the limited feasibility of blinding in exercise trials and the insufficient reporting of several bias domains, rather than to deficiencies in randomisation or attrition handling.

### 3.4 Meta-analysis

#### 3.4.1 Baseline period test

**Figure 2:**
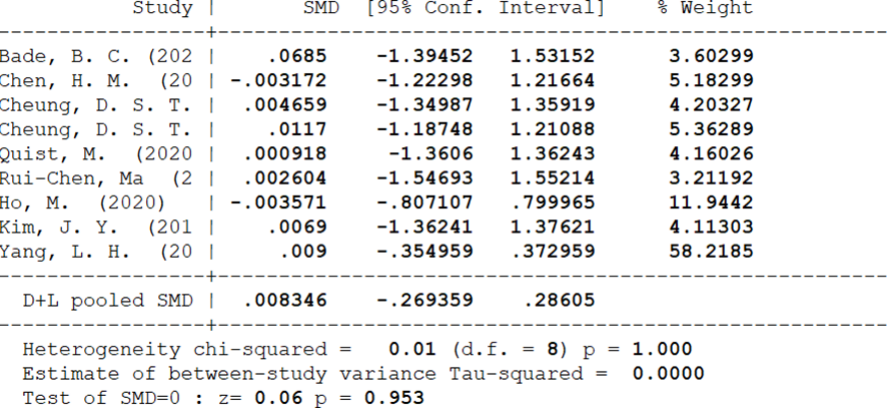
Baseline period test.

Depressive symptom levels were comparable between the exercise and control groups at baseline, with no significant between-group difference observed (SMD = 0.008, 95% CI: -0.269 to 0.286, P = 0.953; **Figure 2**). Between-study heterogeneity was negligible (χ² = 0.01, d.f. = 8, P = 1.000), indicating a high degree of consistency across studies. Yang et al. contributed the largest weight to the pooled estimate (58.22%). These findings indicate that the intervention and control groups were well matched before treatment.

#### 3.4.2 Meta-analysis result

**Figure 3:**
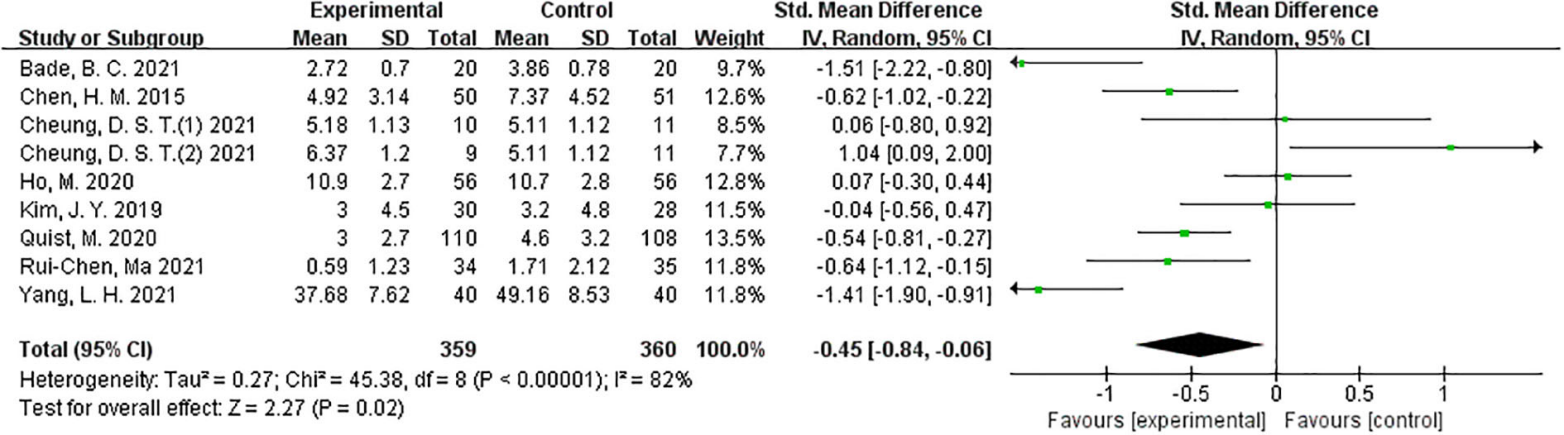
Forest plot of Exercise for Depression.

The pooled meta-analysis showed that exercise interventions were associated with a reduction in depressive symptoms in adults with lung and digestive cancer compared with control conditions (SMD = -0.45, 95% CI: -0.84 to -0.06, P = 0.02; **Figure 3**). The direction of effect favoured the exercise group, suggesting lower post-intervention depression scores among participants receiving exercise-based programmes.

However, substantial between-study heterogeneity was observed (I² = 82%), indicating considerable variability in effect estimates across trials. Therefore, a random-effects model was used. The relatively wide confidence interval and high heterogeneity suggest that the effect of exercise on depressive symptoms may vary according to clinical, methodological, and intervention-related characteristics. These findings should therefore be interpreted cautiously as an overall estimate across heterogeneous study populations and intervention contexts, rather than as definitive evidence of a consistent effect across all adults with lung and digestive cancer.

#### 3.4.3 Publication bias test

**Figure 4:**
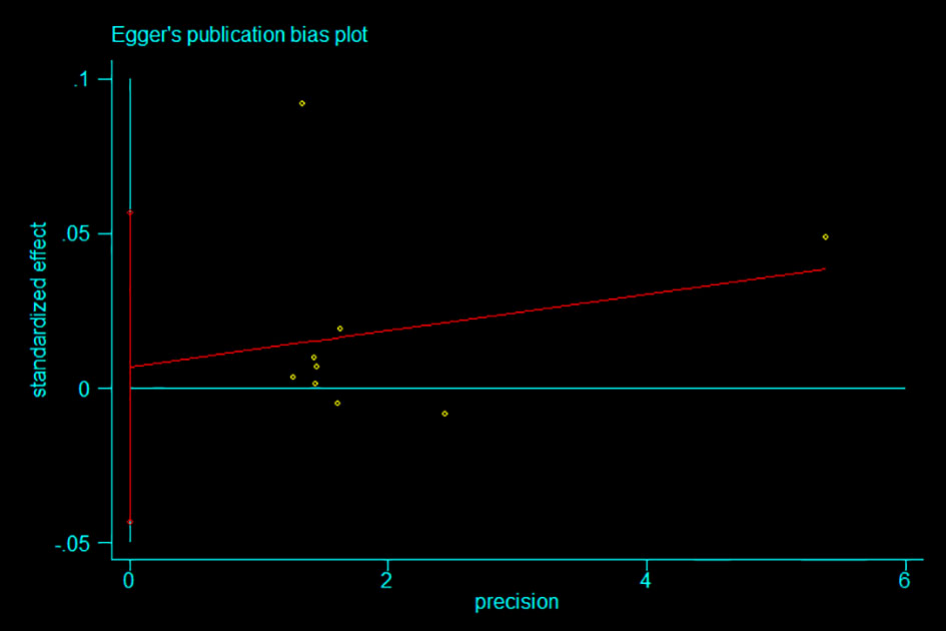
Egger’s publication bias plot.

**Figure 5:**
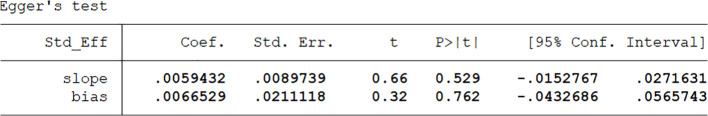
Egger’s test.

Potential publication bias was evaluated by visual inspection of the Egger’s publication bias plot and by Egger’s regression test. As shown in **Figure 4**, the distribution of studies was not markedly asymmetric, and no obvious small-study effect was visually apparent. This impression was supported by the statistical results shown in **Figure 5**. Specifically, Egger’s test did not indicate statistically significant asymmetry (bias coefficient = 0.0067, t = 0.32, P = 0.762), and the slope was likewise non-significant (P = 0.529).

Taken together, the graphical and statistical findings did not provide clear evidence of publication bias in the present meta-analysis. However, because only eight studies were included, the statistical power of Egger’s test was limited. Therefore, publication bias and small-study effects cannot be ruled out, and these findings should be interpreted cautiously.

#### 3.4.4 Subgroup analysis

Given the limited number of included trials, all subgroup analyses were interpreted as exploratory. No statistically significant between-subgroup differences were observed for intervention format, exercise type, or training frequency. Therefore, the subgroup findings should not be considered confirmatory evidence regarding the superiority of any specific exercise programme characteristic.

##### 3.4.4.1 Intervention format

**Figure 6:**
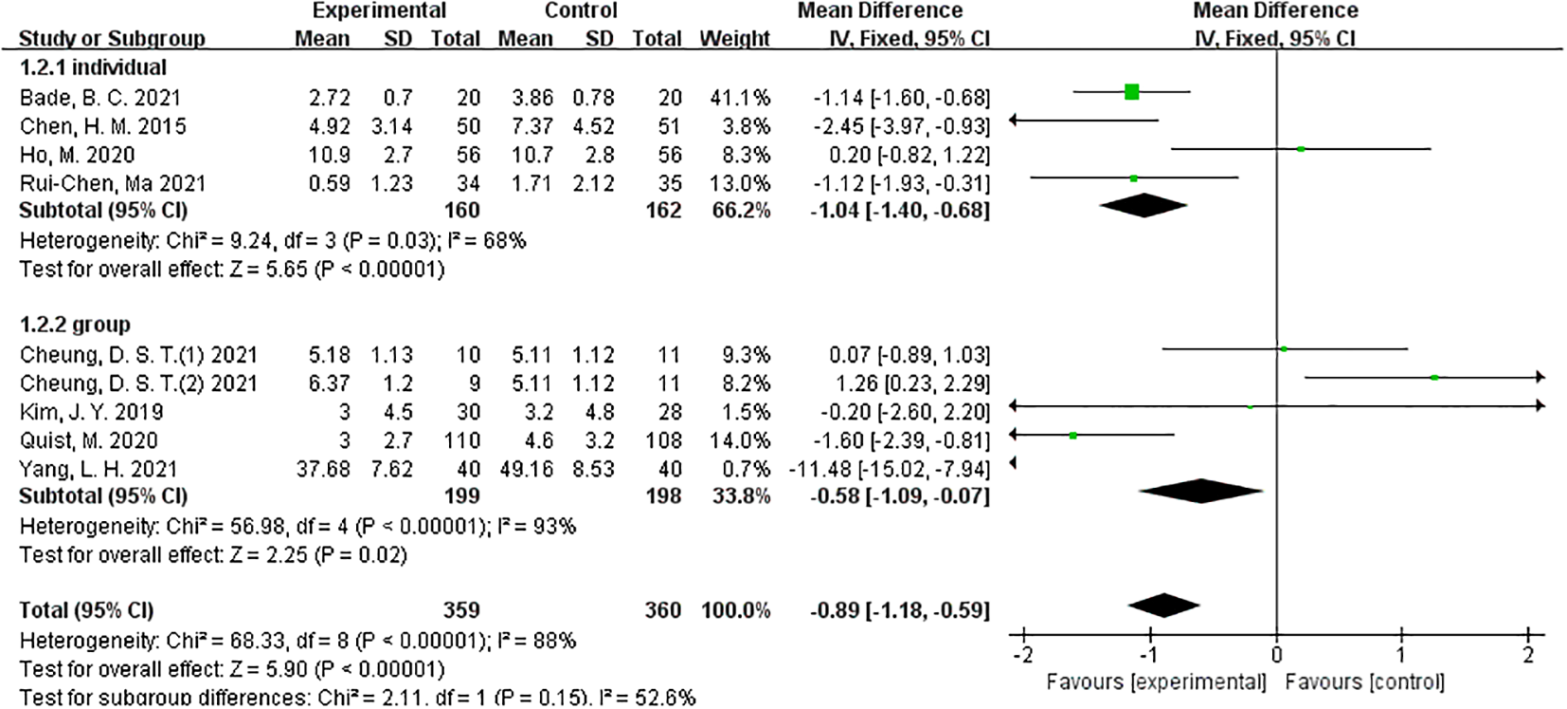
Forest plot of subgroup analyses by individual versus group.

Subgroup analysis by intervention format showed that both individual-based and group-based exercise programmes favoured the exercise group for depressive symptoms, although heterogeneity was moderate to substantial within subgroups (**Figure 6**).

For individual-based programmes, the pooled effect estimate favoured exercise and reached statistical significance. Group-based programmes also showed an effect estimate favouring exercise, with substantial heterogeneity. However, the test for subgroup differences was not statistically significant. Therefore, the numerically larger effect estimate observed for individual-based programmes should be interpreted cautiously and should not be taken as evidence that Individually delivered exercise is superior to group-based exercise.

##### 3.4.4.2 Exercise types

**Figure 7:**
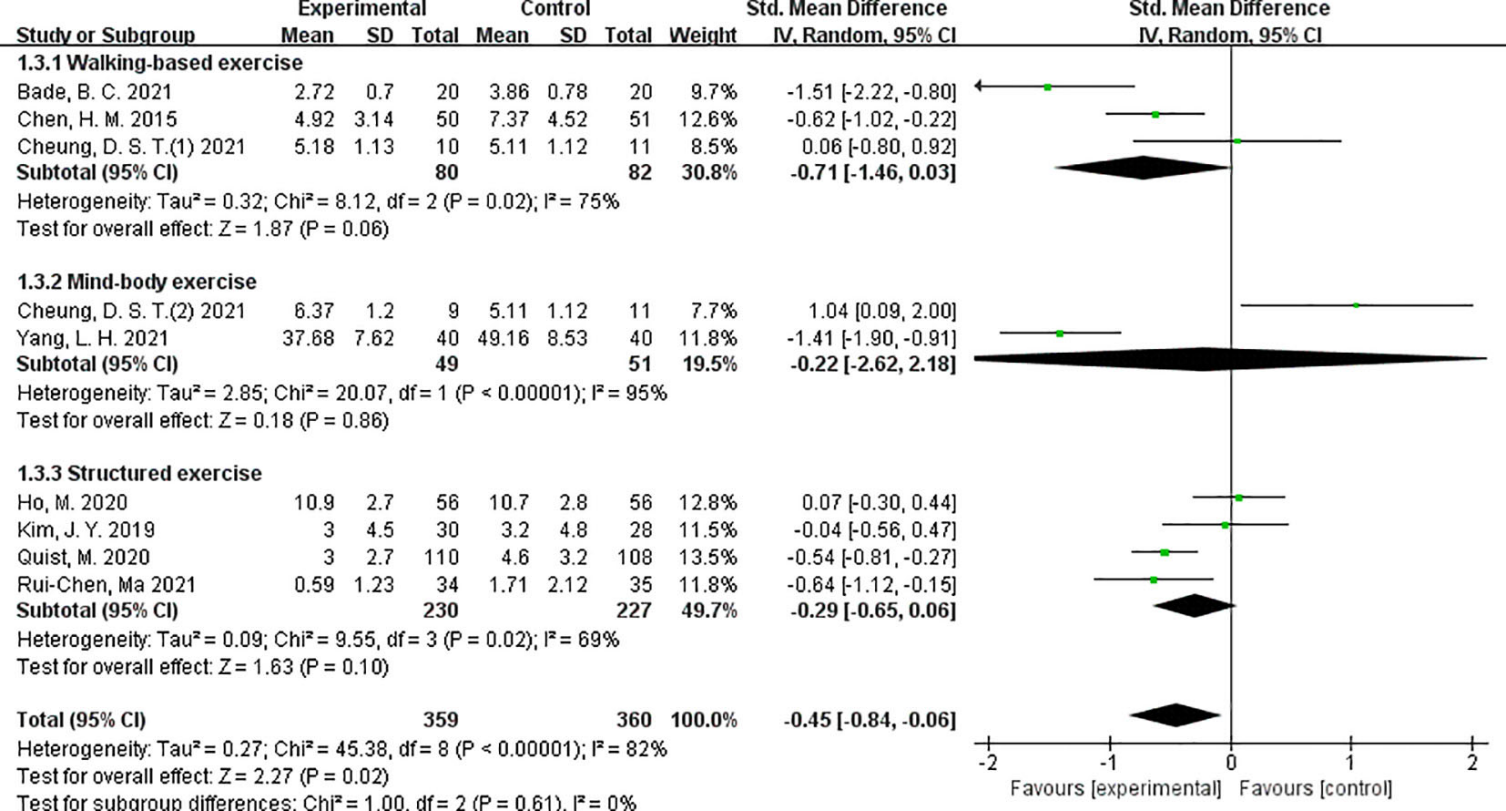
Forest plot of subgroup analyses by type of exercise.

Subgroup analysis by exercise type did not show a statistically significant between-subgroup difference (**Figure 7**). For walking-based exercise, the pooled estimate favoured the exercise group but did not reach statistical significance (P = 0.06), with substantial heterogeneity. For mind–body exercise, no significant pooled effect was observed (P = 0.86), and heterogeneity was very high. Structured exercise also did not show a statistically significant pooled effect (P = 0.10), although the point estimate favoured exercise, with substantial heterogeneity.

Although the pooled effect estimate was numerically largest for walking-based exercise, the absence of a statistically significant between-subgroup difference and the substantial heterogeneity within categories preclude firm conclusions regarding the relative effectiveness of different exercise types.

##### 3.4.4.3 Training frequency

**Figure 8:**
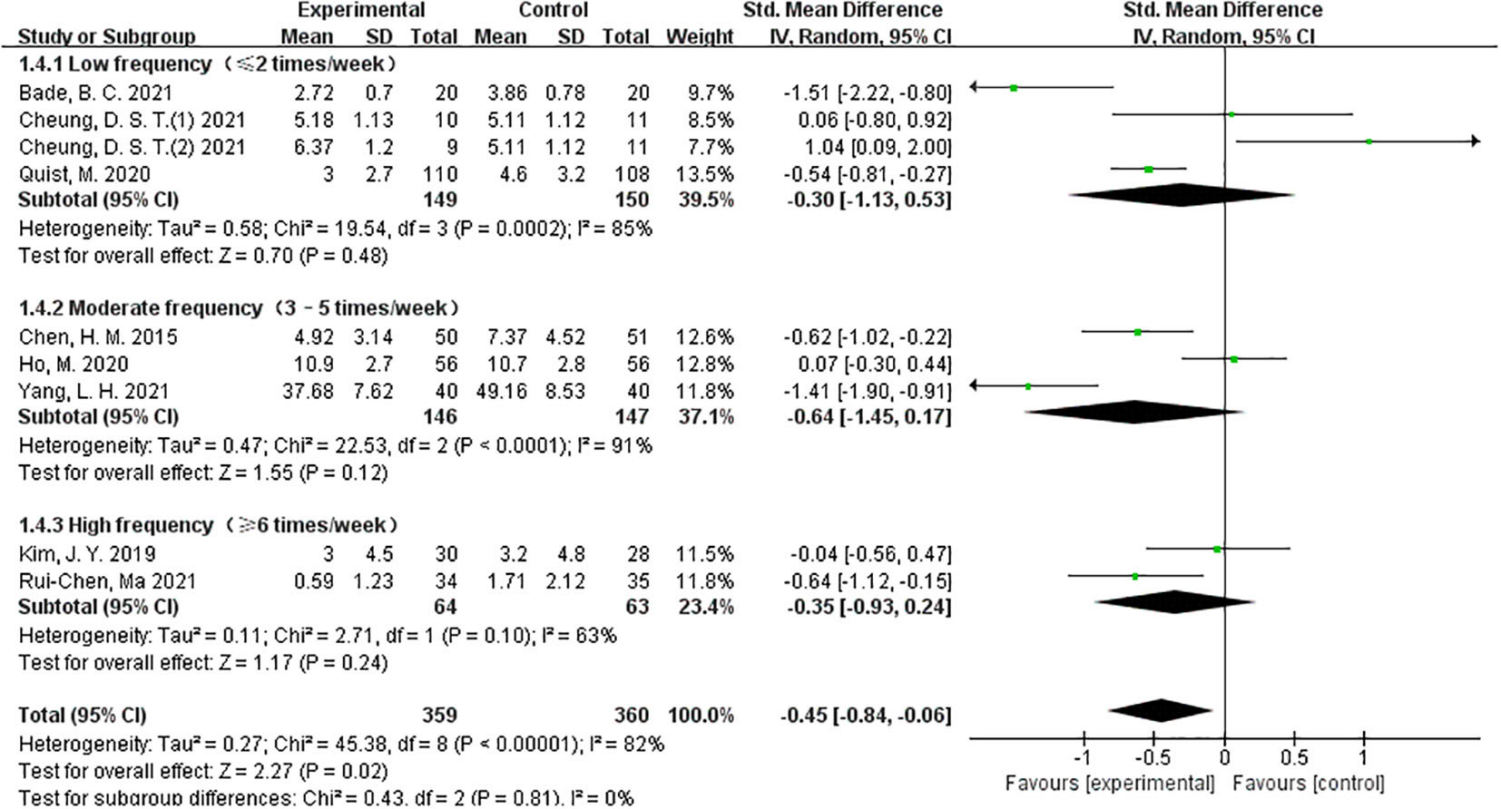
Forest plot of subgroup analyses by training frequency.

Subgroup analysis by training frequency did not show a statistically significant between-subgroup difference (**Figure 8**). For low-frequency programmes (≤2 times/week), the pooled effect estimate favoured exercise but was not statistically significant (P = 0.48), with substantial heterogeneity. For moderate-frequency programmes (3–5 times/week), the pooled estimate also favoured exercise but did not reach statistical significance (P = 0.12), with considerable heterogeneity. For high-frequency programmes (≥6 times/week), the pooled estimate favoured exercise but was not statistically significant (P = 0.24), and heterogeneity was moderate to substantial.

Although moderate-frequency programmes showed the largest numerical effect estimate, this finding should be interpreted cautiously. Given the non-significant test for subgroup differences, the small number of trials within each subgroup, and the persistence of heterogeneity, current evidence is insufficient to determine the optimal weekly training frequency.

### 3.5 Certainty of evidence

The certainty of evidence for the primary outcome was assessed using the GRADE approach (**Table 2**). The certainty of evidence for depressive symptoms was rated as low. The evidence was downgraded mainly because of substantial between-study heterogeneity (I² = 82%) and imprecision related to the small number of included trials and the relatively wide confidence interval. Methodological concerns were also present in some studies.

**Table 2:** GRADE assessment for the primary outcome.

Outcome	No. of studies	Participants	Effect estimate	Certainty of evidence	Reasons for downgrading
Depressive symptoms	8 RCTs	719	SMD=0.45, 95% CI: -0.84 to -0.06	Low	1. Downgraded for inconsistency because substantial heterogeneity was observed (I² = 82%); 2. Downgraded for imprecision because of the small number of trials and relatively wide confidence interval. Methodological concerns were also present in some trials.

## 4 Discussion

The present meta-analysis synthesized evidence from randomized controlled trials and showed that exercise interventions were associated with a reduction in depressive symptoms in adults with lung and digestive cancer. However, substantial heterogeneity was observed in the primary analysis, indicating that the pooled estimate should be interpreted cautiously. This heterogeneity may be attributable to several clinical and methodological factors. First, the included studies involved different cancer populations, including lung cancer and various digestive cancers, which may differ in disease trajectory, treatment burden, symptom profile, and rehabilitation needs. Second, exercise interventions varied in modality, intensity, frequency, duration, supervision, and delivery format. Third, depressive symptoms were assessed using different scales, which may capture different dimensions of psychological distress. In addition, differences in treatment stage, baseline depressive symptom severity, and physical function may have contributed to between-study variability. Therefore, the pooled effect should be interpreted as an overall estimate across heterogeneous clinical and intervention contexts rather than as definitive evidence of a consistent effect across all patients or a single optimal exercise prescription.

The pooled effect size (SMD = -0.45) suggests a small-to-moderate reduction in depressive symptoms. From a clinical perspective, this indicates that exercise interventions may have a supportive role in psychological care for adults with lung and digestive cancer. However, because SMDs are scale-free measures derived from different depression instruments, the effect cannot be directly translated into a specific score reduction on a single clinical scale. Therefore, exercise should be considered as a potentially useful adjunctive strategy rather than a replacement for professional psychological or psychiatric care.

At the subgroup level, no statistically significant differences were observed according to intervention format, exercise type, or training frequency. Therefore, these subgroup findings should be considered exploratory and hypothesis-generating rather than confirmatory. Individually delivered programmes showed a numerically larger pooled effect than group-based interventions, but this difference was not statistically significant and may reflect chance variation or differences in study characteristics. Similarly, walking-based exercise and moderate-frequency programmes appeared to show favourable effect estimates; however, the limited number of trials within each subgroup precludes firm conclusions regarding the superiority of any specific exercise modality or training frequency. These findings may help inform future trial design, but they should not be interpreted as evidence for specific clinical recommendations.

These findings extend the existing literature in several important ways. Earlier evidence syntheses of exercise and depressive symptoms in cancer survivors often pooled multiple cancer types together, which increased clinical heterogeneity and limited the applicability of findings to specific malignancies (21–23). In addition, much of the more focused evidence has come from breast cancer populations, where exercise has been reported to reduce depressive symptoms and improve broader psychosocial outcomes (24–27). More recent cancer-wide reviews and guidelines, including the 2023 ASCO guideline update on anxiety and depression in adult cancer survivors (13), support structured physical activity as one management option, but also reflect the relative scarcity of high-certainty, tumour-specific evidence beyond breast cancer. Against this background, the present study offers a more targeted assessment by focusing specifically on adults with lung and digestive cancer. These groups are clinically important because they often experience substantial symptom burden, treatment-related distress and functional decline, yet they remain underrepresented in exercise-oncology meta-analyses. By restricting inclusion to randomized trials in these two cancer populations, our study reduces some of the clinical heterogeneity inherent in pan-cancer reviews and provides a more directly relevant estimate for these understudied patient groups.

Several biological and behavioural pathways may explain why exercise alleviates depressive symptoms in patients with lung and digestive cancer. From a physiological perspective, exercise may modulate systemic inflammation, improve neuroendocrine regulation and enhance neurotransmitter signalling, all of which have been implicated in the pathophysiology of depression (14). Exercise may also attenuate cancer- and treatment-related fatigue, preserve physical function and improve sleep, thereby interrupting the reciprocal relationship between physical decline and emotional distress. In oncology populations more broadly, exercise has been associated with improvements in symptom burden, functional capacity and health-related quality of life, which may in turn contribute to lower depression scores (28). Behavioural mechanisms are also likely to be relevant. Participation in exercise can enhance self-efficacy, restore a sense of control and promote social interaction, especially during a period often characterised by uncertainty, reduced independence and treatment-related disruption (29). For patients with lung and digestive cancer, these effects may be particularly meaningful because breathlessness, gastrointestinal symptoms, nutritional impairment and overall deconditioning can markedly restrict normal daily activities and reinforce psychological vulnerability.

This study should also be interpreted in light of several limitations. First, the number of included trials was relatively small, which limited statistical power and reduced the precision of subgroup analyses. Second, substantial heterogeneity was observed in the main pooled analysis and within several subgroups, indicating important variation in intervention content, participant characteristics, treatment stage, and outcome assessment. The high level of heterogeneity limits the certainty and clinical interpretability of the pooled estimate. In particular, the digestive cancer category included clinically diverse malignancies, such as colorectal, gastric, oesophageal, hepatic, pancreatic, and other gastrointestinal cancers, which may differ substantially in disease trajectory, treatment burden, nutritional status, symptom profile, functional impairment, and rehabilitation needs. Pooling these cancers may therefore reduce the specificity and clinical interpretability of the findings. Because of the limited number of eligible trials, cancer-specific subgroup analyses within digestive cancers could not be conducted reliably. Although exploratory subgroup and sensitivity analyses were conducted, the small number of included trials limited our ability to fully identify the sources of heterogeneity. Third, depressive symptoms were measured using different instruments across studies, requiring the use of standardised mean differences; although methodologically appropriate, this may reduce clinical interpretability. Fourth, blinding of participants and personnel was universally difficult in exercise trials, which may have introduced performance bias, particularly for self-reported psychological outcomes. In addition, the risk-of-bias assessment was conducted using the Cochrane RoB 1 tool rather than RoB 2. Although RoB 1 provides a transparent domain-based assessment and remains widely used in exercise-intervention reviews, RoB 2 may provide a more outcome-specific evaluation of bias in randomized trials. Finally, publication bias could not be reliably assessed because fewer than ten studies were included. Although neither the funnel plot nor Egger’s test suggested clear publication bias, these methods have limited power when applied to such a small number of studies. Therefore, the possibility of small-study effects or unpublished negative findings cannot be excluded. Taken together, the present findings suggest that exercise interventions may be associated with reductions in depressive symptoms in adults with lung and digestive cancer, but the small evidence base, substantial heterogeneity, and limited certainty of evidence indicate that these findings should be interpreted cautiously. Larger, methodologically rigorous trials are needed to clarify the optimal exercise prescription and identify the populations most likely to benefit.

## 5 Conclusion

In conclusion, this meta-analysis of randomized controlled trials indicates that exercise interventions are associated with a significant reduction in depressive symptoms in adults with lung and digestive cancer. Although subgroup differences were not statistically significant, Individually delivered programmes, walking-based exercise and moderate-frequency training showed numerically larger effect estimates, suggesting potentially favourable directions for future intervention design. These findings extend the current evidence base beyond the predominantly breast cancer-focused literature and support exercise as a promising adjunctive strategy for psychological care in these understudied cancer populations. Further large-scale, high-quality trials are needed to confirm these findings and to define the optimal exercise prescription for reducing depressive symptoms in adults with lung and digestive cancer.

## Data Availability

The original contributions presented in the study are included in the article/**Supplementary Material**. Further inquiries can be directed to the corresponding authors.
