# Supplementary material for: Exercise interventions for depressive symptoms in adults with lung and digestive cancer: a meta-analysis of randomized controlled trials
Source: Front Psychiatry. 2026 Jun 1;17:1833619. doi: 10.3389/fpsyt.2026.1833619 (PMC13265490; doi:10.3389/fpsyt.2026.1833619)
Supplement: Supplementary file 1 [file DataSheet1.pdf]

	Random sequence generation (selection bias)	Allocation concealment (selection bias)	Blinding of participants and personnel (performance bias)	Blinding of outcome assessment (detection bias)	Incomplete outcome data (attrition bias)	Selective reporting (reporting bias)	Other bias
Bade, B. C. 2021	+	?	−	−	+	+	?
Chen, H. M. 2015	+	+	−	−	+	?	?
Cheung, D. S. T.(1) 2021	+	+	−	+	+	+	?
Cheung, D. S. T.(2) 2021							
Ho, M. 2020	+	+	−	−	+	+	?
Kim, J. Y. 2019	+	?	−	?	+	?	?
Quist, M. 2020	+	+	−	?	+	+	+
Rui-Chen, Ma 2021	+	+	−	+	+	?	?
Yang, L. H. 2021	+	+	−	+	+	?	?
